# Prognosis and treatment outcomes for patients with stage IA triple-negative breast cancer

**DOI:** 10.1038/s41523-024-00634-6

**Published:** 2024-04-04

**Authors:** Paolo Tarantino, Julieta Leone, Carlos T. Vallejo, Rachel A. Freedman, Adrienne G. Waks, Olga Martínez-Sáez, Ana Garrido-Castro, Filipa Lynce, Nabihah Tayob, Nancy U. Lin, Sara M. Tolaney, Jose P. Leone

**Affiliations:** 1 Department of Medical Oncology, Dana-Farber Cancer Institute, Boston, MA USA; 2 Breast Oncology Program, Dana-Farber Brigham Cancer Center, Boston, MA USA; 3 Harvard Medical School, Boston, MA USA; 4 Department of Oncology and Onco-Hematology, University of Milan, Milan, Italy; 5 Grupo Oncológico Cooperativo del Sur, Neuquén, Argentina; 6 Medical Oncology Department, Hospital Clinic of Barcelona, Barcelona, Spain; 7 Translational Genomics and Targeted Therapeutics in Solid Tumors, August Pi i Sunyer Biomedical Research Institute (IDIBAPS), Barcelona, Spain

**Keywords:** Breast cancer, Breast cancer

## Abstract

To evaluate the role of chemotherapy in stage IA triple-negative breast cancer, we conducted a retrospective population-based study including 8601 patients. The use of chemotherapy significantly increased from 2010 to 2019 in patients with T1b and T1c tumors (*p* = 0.001 and *p* < 0.001, respectively). Receipt of chemotherapy was associated with improved breast cancer-specific survival (BCSS, adjusted hazard ratio = 0.70; *p* = 0.006), particularly in patients with T1c tumors (5-year BCSS 94.5% vs. 91.2%).

Patients with early-stage triple-negative breast cancer (TNBC) have a higher risk of distant recurrence and death compared to patients with other breast cancer subtypes, including patients with small tumors^1^. Currently, adjuvant chemotherapy is the mainstay of systemic therapy for patients with stage I TNBC, who represent approximately one-third of TNBC patients.2 Among patients with stage IA TNBC (i.e., tumors ≤2 cm and node-negative), the National Comprehensive Cancer Network guidelines recommend adjuvant chemotherapy for tumors 1.1–2 cm (T1c), with consideration of adjuvant chemotherapy for tumors 0.6–1 cm (T1b). Patients with tumors ≤0.5 cm (T1a) are not recommended to receive adjuvant chemotherapy^2,3^. However, the utilization and benefit of chemotherapy for this population in the modern era remain poorly defined.

We conducted a population-based study using Surveillance, Epidemiology, and End Results (SEER) to investigate adjuvant chemotherapy treatment patterns and survival outcomes among patients diagnosed with stage IA TNBC between 2010 and 2019. We included 8601 women diagnosed with stage IA TNBC between 2010 and 2019. Patient demographics and disease information are included in Table 1. The median age at diagnosis was 62 years old. Most patients (92.79%) had invasive ductal carcinomas, T1c disease (60.85%) and grade 3 (i.e., high-grade) tumors (70.14%). Adjuvant chemotherapy was administered to 5295 patients (61.6%). Median follow-up for survival analyses was 48 months (interquartile range: 20–83 months).

**Table 1:** Patient characteristics

All Patients (*n* = 8601)	Chemotherapy	
	No/Unknown (*n* = 3306)	Yes (*n* = 5295)
Age at diagnosis (years)		
<50	323 (9.77%)	1200 (22.66%)
50–64	992 (30.01%)	2486 (46.95%)
>64	1991 (60.22%)	1609 (30.39%)
Race		
Non-Hispanic White	2137 (64.64%)	3326 (62.81%)
Non-Hispanic Black	518 (15.67%)	916 (17.30%)
Non-Hispanic American	14 (0.42%)	28 (0.53%)
Indian/Alaska Native		
Non-Hispanic Asian or Pacific Islander	275 (8.32%)	393 (7.42%)
Hispanic (All Races)	339 (10.25%)	610 (11.52%)
Unknown	23 (0.70%)	22 (0.42%)
Marital status at diagnosis		
Single	406 (12.28%)	744 (14.05%)
Married	1618 (48.94%)	3197 (60.38%)
Domestic Partner	10 (0.30%)	16 (0.30%)
Other (Separated/Divorced/Widowed)	1076 (32.55%)	1097 (20.72%)
Unknown	196 (5.93%)	241 (4.55%)
Median household income		
≥$75,000	978 (29.58%)	1762 (33.28%)
$65,000–$74,999	708 (21.42%)	1090 (20.59%)
$55,000–$64,999	741 (22.41%)	1021 (19.28%)
$45,000–$54,999	534 (16.15%)	804 (15.18%)
$35,000–$44,999	276 (8.35%)	498 (9.40%)
<$35,000	68 (2.06%)	119 (2.25%)
Unknown	1 (0.03%)	1 (0.02%)
Rural/Urban		
Metropolitan areas of ≥1,000,000 population	1885 (57.02%)	2968 (56.05%)
Metropolitan areas of 250,000–1,000,000 population	703 (21.26%)	1160 (21.91%)
Metropolitan areas of <250,000 population	307 (9.29%)	450 (8.50%)
Metropolitan adjacent to a metropolitan area	249 (7.53%)	440 (8.31%)
Metropolitan not adjacent to a metropolitan area	157 (4.75%)	267 (5.04%)
Unknown	5 (0.15%)	10 (0.19%)
Histology		
Ductal	2987 (90.35%)	4994 (94.32%)
Lobular	43 (1.30%)	33 (0.62%)
Ductal and lobular	22 (0.67%)	40 (0.76%)
Other	254 (7.68%)	228 (4.30%)
Grade		
I (well differentiated)	256 (7.74%)	89 (1.68%)
II (moderately differentiated)	1041 (31.49%)	980 (18.51%)
III/IV (poorly differentiated/anaplastic)	1875 (56.72%)	4158 (78.53%)
Unknown	134 (4.05%)	68 (1.28%)
T		
T1mic	210 (6.35%)	22 (0.42%)
T1a	744 (22.50%)	216 (4.08%)
T1b	863 (26.10%)	1312 (24.78%)
T1c	1489 (45.04%)	3745 (70.73%)
Surgery		
Partial Mastectomy	2367 (71.60%)	3820 (72.14%)
Mastectomy	939 (28.40%)	1475 (27.86%)
Radiation Therapy		
No/unknown	1643 (49.70%)	2024 (38.22%)
Yes	1663 (50.30%)	3271 (61.78%)
Status		
Alive	2899 (87.69%)	5028 (94.96%)
Dead	407 (12.31%)	267 (5.04%)
Cause of Death		
Alive	2899 (87.69%)	5028 (94.96%)
Dead from breast cancer	141 (4.26%)	185 (3.49%)
Dead from other causes	266 (8.05%)	82 (1.55%)

The rate of chemotherapy use among patients with T1mic and T1a tumors did not change significantly between 2010 and 2019 (<20% and <30% across years, respectively). However, chemotherapy use increased significantly from 2010-2019 among patients with T1b (*p* = 0.001) and T1c tumors (*p* < 0.0001), reaching ≥60% in patients with T1b and ≥70% in patients with T1c tumors across most years (Supplementary Table 1, Supplementary Fig. 1).

In multivariable analyses, variables significantly associated with chemotherapy use (all *p* < 0.02) were younger age (age <50 vs. >64, odds ratio [OR] = 5.19), married status (married vs. single, OR = 1.28), high tumor grade (high grade [grade III] vs. low grade [grade I], OR = 4.89), and tumor size (Reference T1mic: T1a, OR = 2.91; T1b, OR = 19.16; T1c, OR = 31.49), among others (Supplementary Table 2).

Multivariable analyses revealed that chemotherapy receipt (vs. no/unknown receipt) was associated with improved BCSS among patients with stage IA TNBC. The 5-year BCSS for patients receiving chemotherapy vs. no/unknown was 95.2% vs. 94.4% (Adjusted hazard ratio [HR] = 0.70; 95% confidence interval [CI]: 0.55–0.90; Cox *p* value = 0.006) (Supplementary Fig. 2). When BCSS was evaluated by tumor size, patients with T1mic (Fig. 1a) and T1a (Fig. 1b) had excellent outcomes regardless of chemotherapy administration, with finding of only 1 (0.4%) and 17 (1.8%) breast cancer deaths, respectively (Supplementary Table 3). The small number of events in these two groups prevented adjusted comparisons. For those with T1b cancers, there was no significant association between chemotherapy and BCSS, with a total of 63 (2.9%) breast-cancer deaths observed (Fig. 1c). In the setting of T1c disease, a total of 245 (4.7%) breast-cancer deaths were observed; in this subgroup, chemotherapy was associated with improved BCSS, with a 5-year BCSS of 94.5% for patients receiving chemotherapy vs. 91.2% in the no/unknown chemotherapy group (Adjusted HR = 0.64; 95% CI: 0.48–0.85; Cox *p* value = 0.002) (Fig. 1d).

**Fig. 1:**
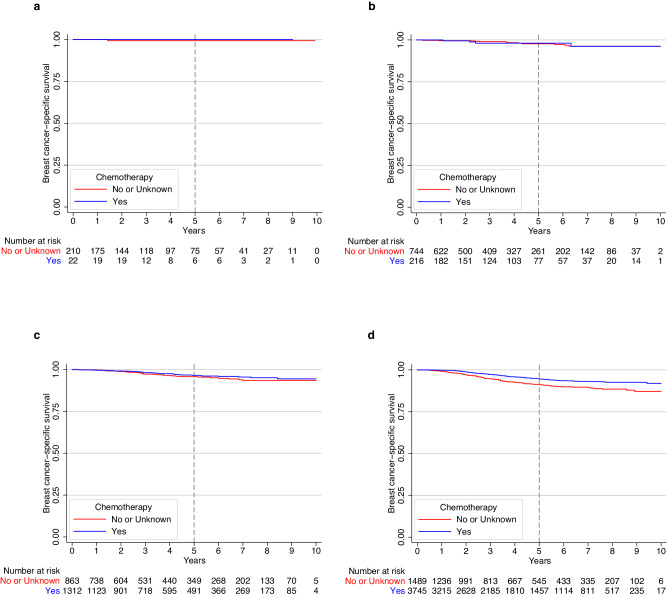
Breast cancer-specific survival (BCSS) in women with stage IA triple-negative breast cancer. BCSS in **a**) patients with T1mic disease; **b**) patients with T1a disease; **c**) patients with T1b disease; and **d**) patients with T1c disease.

Our large population-based study revealed that women with stage IA TNBC have excellent 5-year BCSS outcomes, and that associations of chemotherapy receipt with survival were primarily observed in patients with T1c disease. Our findings are relatively consistent with similar studies conducted in this space, despite different sample sizes and methodologic differences. Vaz-Luis et al.^4^ analyzed outcomes of patients with T1a-T1bN0 TNBC in the NCCN database (*n* = 363), highlighting favorable 5-year BCSS ( ≥ 95%) in both T1a and T1b, irrespective of chemotherapy receipt. Studies conducted in SEER by refs. ^5–7^ showed improved outcomes among patients with T1c TNBC who received chemotherapy. In contrast to our study, the above studies included patients treated in the neoadjuvant setting, potentially including patients with occult nodal disease; we opted for excluding this population, so that we could ensure inclusion of patients with node-negative disease and provide more reliable estimates. Additionally, our study evaluated outcomes adjusted for histology, race, ethnicity, marital status, income, and rurality, which are relevant prognostic factors. A recent study by Carbajal-Ochoa evaluated only T1b and T1c TNBC and showed improved BCSS in T1c tumors^8^.

Our study adds to a body of evidence that suggests a benefit of adjuvant chemotherapy among stage IA TNBC patients with T1c tumors. Additionally, we observed an increase in chemotherapy use over time among patients with T1b and T1c tumors, possibly associated with increased reporting on recurrence rates for these tumors. Our study also provides important information on the favorable outcomes of patients with tumors ≤1 cm (T1mic, T1a, and T1b) that can inform discussions with patients. Indeed, the limited data available to treat small TNBCs has commonly led to extrapolation from studies of larger TNBCs for treating patients in clinical practice, with relevant risk for overtreatment and unnecessary toxicity. Important limitations of our study include its retrospective nature, with potential prognostic imbalances between subgroups, the absence of recurrence data in SEER and the lack of information on patient/clinician preferences and type of chemotherapies administered. Of note, the chemotherapy variable is coded in SEER as “no/unknown” when there was no evidence of chemotherapy administration, which prevents us from separating “no” from “unknown”.

In conclusion, in a large population-based study we observed excellent long-term outcomes for patients with stage IA TNBC and identified a progressive increase in the use of adjuvant chemotherapy for this population. An association between BCSS and use of adjuvant chemotherapy was identified for patients with T1c tumors, but not for T1b. Integration of additional prognostic factors and shared decision-making for treatment choices in this low-risk setting are warranted.

## Methods

### Data source and study design

We obtained data from SEER, using the 17 registries database (Nov. 2021 Submission). We extracted all cases of women diagnosed with stage IA TNBC (T1mic,a,b,c,N0,M0) from 2010 to 2019. To be included, patients must have had only one primary malignancy in their lifetime, had known vital status and cause of death (*n* = 10,048). We excluded patients who did not undergo definitive breast surgery (*n* = 314), those who received neoadjuvant chemotherapy (*n* = 1116) or neoadjuvant radiation therapy (*n* = 17) (Supplementary Fig. 3). The following variables were collected for analysis: age at diagnosis, race and ethnicity, marital status, median household income, rurality, year of diagnosis, histology, tumor grade, stage and size (*T*), type of surgery, receipt of adjuvant chemotherapy (coded as yes vs. no/unknown), receipt of adjuvant radiation therapy, vital status, and cause of death.

### Statistical analyses

We examined the cohort characteristics and the frequency of chemotherapy use over time by tumor size. Variables associated with receipt of chemotherapy were evaluated using multivariate logistic regression. Nonparametric test for trend was used to evaluate the trend in chemotherapy use over time. We also examined breast cancer-specific survival (BCSS), defined as the interval from initial breast cancer diagnosis to death from breast cancer or last follow-up for censored patients. We used multivariable cox models to evaluate the association of adjuvant chemotherapy with BCSS stratified by tumor size, adjusted for age, race, ethnicity, tumor grade, histology, receipt of radiation therapy, marital status, income, and rurality. All *p* values were two-tailed and values <0.05 were considered statistically significant.

### Ethical considerations

This study used de-identified, publicly available data. As such, it was exempt from review by the Dana-Farber Office for Human Research Studies.

### Reporting summary

Further information on research design is available in the Nature Research Reporting Summary linked to this article.

### Supplementary information

Supplementary Tables and Figures

Reporting Summary

## Data Availability

Jose P. Leone had full access to all of the data in the study and took responsibility for the integrity of the data and the accuracy of the data analysis. The data that support the findings of this study are publicly available at SEER: https://seer.cancer.gov/.
