# Supplementary material for: Prognosis and treatment outcomes for patients with stage IA triple-negative breast cancer
Source: NPJ Breast Cancer. 2024 Apr 4;10:26. doi: 10.1038/s41523-024-00634-6 (PMC10995121; doi:10.1038/s41523-024-00634-6)
Supplement: Supplementary file 1 — Supplementary Tables and Figures [file 41523_2024_634_MOESM1_ESM.pdf]

Supplementary Table 1.
Chemotherapy use over time, stratified by tumor size

T1mic (n = 232)		
**Year**	**No/Unknown Chemo**	**Yes Chemo**
2010	11 (100%)	0 (0%)
2011	16 (88.9%)	2 (11.1%)
2012	18 (94.7%)	1 (5.3%)
2013	14 (82.4%)	3 (17.6%)
2014	19 (100%)	0 (0%)
2015	24 (96%)	1 (4%)
2016	21 (80.8%)	5 (19.2%)
2017	26 (81.3%)	6 (18.8%)
2018	28 (96.6%)	1 (3.4%)
2019	33 (91.7%)	3 (8.3%)
**Total**	210 (90.52%)	22 (9.48%)
p-value for trend	0.567	
**T1a (n = 960)**		
**Year**	**No/Unknown Chemo**	**Yes Chemo**
2010	43 (76.8%)	13 (23.2%)
2011	60 (89.6%)	7 (10.4%)
2012	66 (79.5%)	17 (20.5%)
2013	66 (76.7%)	20 (23.3%)
2014	55 (70.5%)	23 (29.5%)
2015	59 (73.8%)	21 (26.3%)
2016	76 (75.2%)	25 (24.8%)
2017	91 (74.6%)	31 (25.4%)
2018	111 (78.7%)	30 (21.3%)
2019	117 (80.1%)	29 (19.9%)
**Total**	744 (77.5%)	216 (22.5%)
p-value for trend	0.637	
**T1b (n = 2175)**		
**Year**	**No/Unknown Chemo**	**Yes Chemo**
2010	87 (47.5%)	96 (52.5%)
2011	81 (45.3%)	98 (54.7%)
2012	78 (43.1%0	103 (56.9%)
2013	67 (39.9%)	101 (60.1%)
2014	82 (40.6%)	120 (59.4%)
2015	91 (44.2%)	115 (55.8%)
2016	79 (39.1%)	123 (60.9%)
2017	58 (25.7%)	168 (74.3%)
2018	122 (37.1%)	207 (62.9%)
2019	118 (39.5%)	181 (60.5%)
**Total**	863 (39.68%)	1312 (60.32%)
p-value for trend	0.001	
**T1c (n = 5234)**		

**Year**	**No/Unknown Chemo**	**Yes Chemo**
2010	164 (37%)	279 (63%)
2011	147 (32.1%)	311 (67.9%)
2012	169 (32.6%)	349 (67.4%)
2013	118 (26.2%)	333 (73.8%)
2014	108 (23.7%)	347 (76.3%)
2015	129 (27%)	348 (73%)
2016	131 (27.4%)	347 (72.6%)
2017	129 (24.3%)	402 (75.7%)
2018	190 (26.5%)	528 (73.5%)
2019	204 (28.9%)	501 (71.1%)
**Total**	1489 (28.45%)	3745 (71.55%)
p-value for trend	< 0.001	

Abbreviations: **Chemo,** chemotherapy

Supplementary Table 2.
Multivariate logistic regression for factors associated with use of chemotherapy

	p-value	Odds Ratio (95% CI)
Age at diagnosis, years		
<50	<0.001	5.191 (4.406 – 6.117)
50-64	<0.001	3.647 (3.253 – 4.089)
>64		Reference
Race/Ethnicity		
Non-Hispanic White		Reference
Non-Hispanic Black	0.630	1.037 (0.894 – 1.203)
Non-Hispanic American Indian/Alaska Native	0.937	0.965 (0.402 – 2.319)
Non-Hispanic Asian or Pacific Islander	0.023	0.795 (0.653 – 0.969)
Hispanic – All Races	0.019	0.816 (0.688 – 0.967)
Unknown	0.016	0.434 (0.220 – 0.858)
Year of Diagnosis		
Each Additional Year	<0.001	1.065 (1.046 – 1.084)
Grade		
I		Reference
II	<0.001	2.860 (2.145 – 3.813)
III/IV	<0.001	4.893 (3.709 – 6.465)
Unknown	<0.001	2.360 (1.502 – 3.710)
Histology		
Ductal		Reference
Lobular	0.379	0.791 (0.469 – 1.334)
Ductal and Lobular	0.644	1.149 (0.638 – 2.067)
Other	<0.001	0.487 (0.393 – 0.604)
T		
T1mic		Reference
T1a	<0.001	2.919 (1.794 – 4.749)
T1b	<0.001	19.164 (11.961 – 30.706)
T1c	<0.001	31.499 (19.743 – 50.255)
Marital status at diagnosis		
Single		Reference
Married	0.003	1.280 (1.090 – 1.504)
Domestic partner	0.726	1.186 (0.457 – 3.077)
Other (Separated/Divorced/Widowed)	0.005	0.780 (0.655 – 0.929)
Unknown	0.037	0.759 (0.585 – 0.983)
Median household income		
≥ $75,000		Reference
$65,000 - $74,999	0.003	0.804 (0.696 – 0.930)
$55,000 - $64,999	<0.001	0.692 (0.596 – 0.804)
$45,000 - $54,999	0.004	0.766 (0.638 – 0.919)
$35,000 - $44,999	0.415	0.906 (0.714 – 1.149)
< $35,000	0.750	0.934 (0.613 – 1.422)
Unknown	0.889	1.731 (0.001 – 3942.344)
Rural/Urban		
Metropolitan areas with population ≥ 1,000,000		Reference
Metropolitan areas with population of 250,000 – 1,000,000	0.204	1.090 (0.954 – 1.246)
Metropolitan areas with population < 250,000	0.753	1.034 (0.841 – 1.271)

Metropolitan adjacent to metropolitan area	0.528	1.077 (0.856 – 1.355)
Nonmetropolitan not adjacent to a metropolitan area	0.851	1.028 (0.773 – 1.367)
Unknown	0.951	1.049 (0.231 – 4.766)

BCSS, breast cancer-specific survival; **CI,** confidence interval

Supplementary Table 3.
BCSS at 3, 5 and 7 years according to tumor size and receipt of chemotherapy

	Stage 1					
	Chemotherapy					
	No/Unk	95% CI		Yes	95% CI	
N	3,306			5,295		
BCSS rate at 3 years	0.966	0.9578	0.9727	0.975	0.9694	0.9796
BCSS rate at 5 years	0.9435	0.9324	0.9529	0.9516	0.9434	0.9587
BCSS rate at 7 years	0.926	0.912	0.9379	0.9375	0.9273	0.9463
	T1mic					
	Chemotherapy					
	No/Unk	95% CI		Yes	95% CI	
N	210			22		
BCSS rate at 3 years	0.9938	0.9565	0.9991	1	NE	NE
BCSS rate at 5 years	0.9938	0.9565	0.9991	1	NE	NE
BCSS rate at 7 years	0.9938	0.9565	0.9991	1	NE	NE
	T1a					
	Chemotherapy					
	No/Unk	95% CI		Yes	95% CI	
N	744				216	
BCSS rate at 3 years	0.9892	0.9756	0.9952	0.9805	0.9404	0.9937
BCSS rate at 5 years	0.977	0.9566	0.9879	0.9805	0.9404	0.9937
BCSS rate at 7 years	0.9627	0.9337	0.9792	0.9617	0.886	0.9875
	T1b					
	Chemotherapy					
	No/Unk	95% CI		Yes	95% CI	
N	863				1312	
BCSS rate at 3 years	0.9726	0.9562	0.983	0.9819	0.9708	0.9888
BCSS rate at 5 years	0.9576	0.9371	0.9716	0.9656	0.9496	0.9766
BCSS rate at 7 years	0.9349	0.9053	0.9555	0.9553	0.9353	0.9692
	T1c					
	Chemotherapy					
	No/Unk	95% CI		Yes	95% CI	
N	1489				3745	
BCSS rate at 3 years	0.9469	0.9314	0.9589	0.9722	0.9651	0.9778
BCSS rate at 5 years	0.9118	0.8911	0.9287	0.9451	0.9347	0.9539
BCSS rate at 7 years	0.8939	0.87	0.9137	0.9299	0.9172	0.9407

Supplementary Figure 1.

Rates of chemotherapy administration by year and tumor size

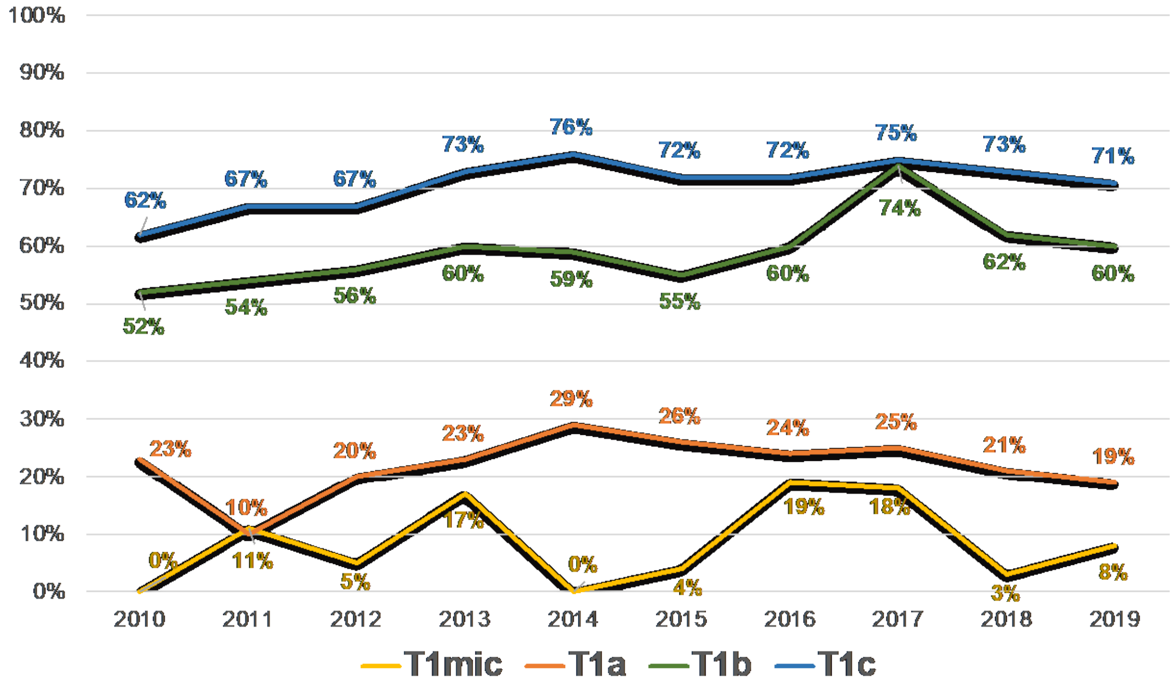

Supplementary Figure 2.
Breast cancer-specific survival for Stage 1A Triple-negative breast cancer

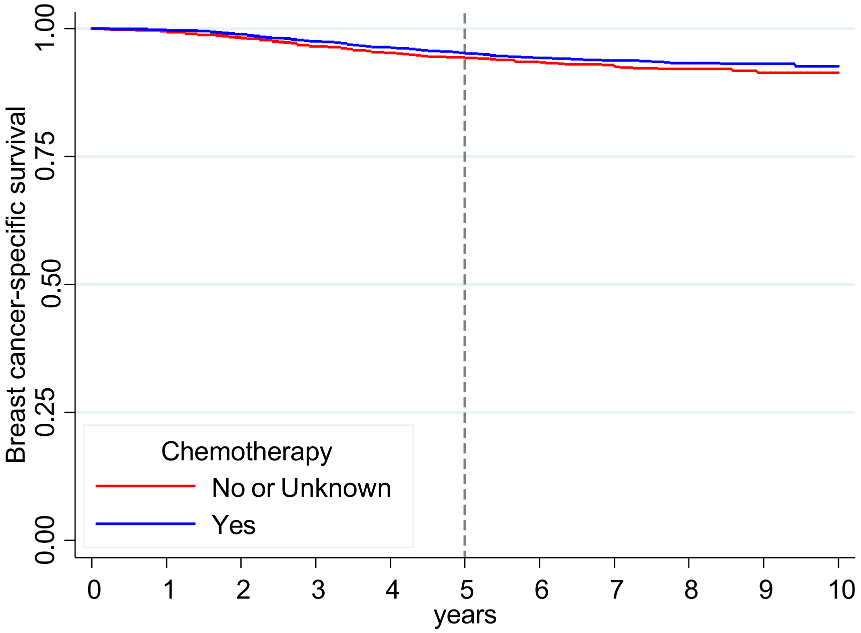

Number at risk

	0	1	2	3	4	5	6	7	8	9	10
No or Unknown	3306	2771	2239	1871	1531	1230	960	720	453	220	13
Yes	5295	4539	3699	3039	2516	2031	1543	1120	712	335	22

	No/Unknown Chemotherapy	Yes Chemotherapy	Adjusted HR	Adjusted p-value
**5-year BCSS (95% CI)**	94.4% (93.2% - 95.3%)	95.2% (94.3% - 95.9%)	0.70 (0.55 – 0.90)	0.006

BCSS, breast cancer-specific survival; **CI,** confidence interval; **HR,** hazard ratio

Supplementary Figure 3.
Selection of patients with stage IA TNBC from SEER.

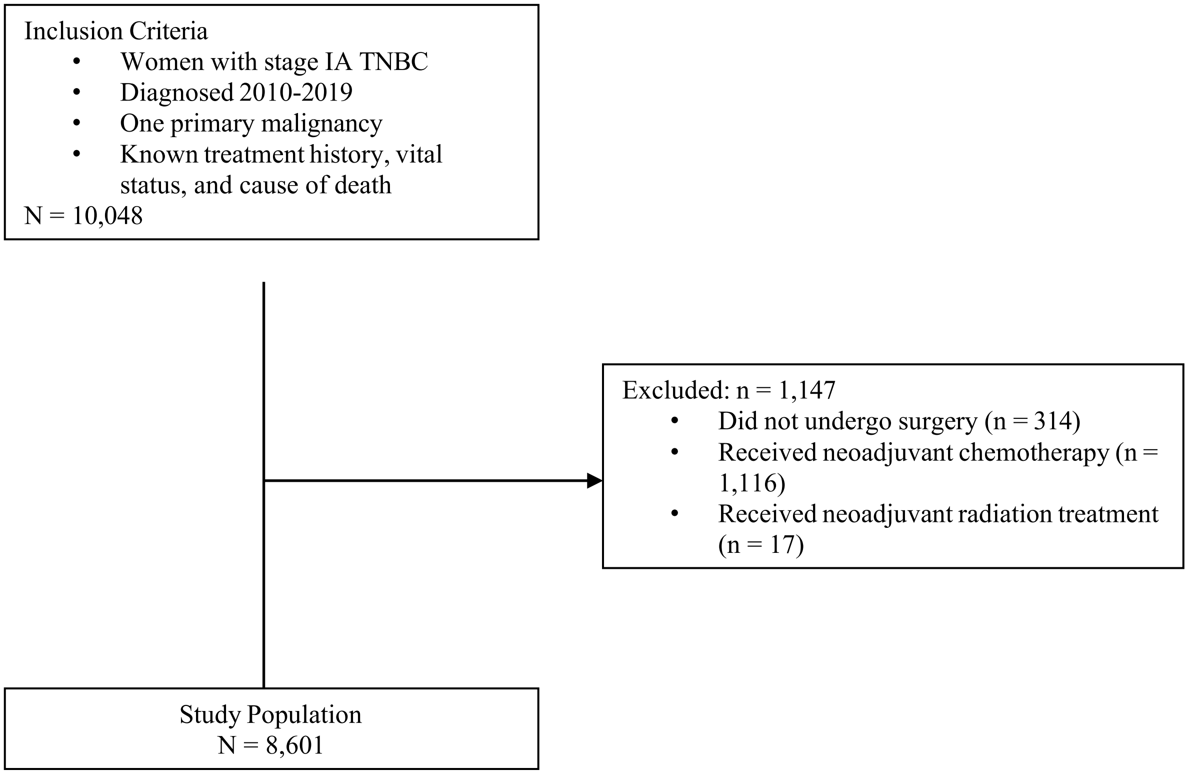

TNBC, triple-negative breast cancer; **SEER,** Surveillance, Epidemiology, and End Results program
